# African leaders take action on RHD: The 4^th^ All-Africa Workshop on Acute Rheumatic Fever and Rheumatic Heart Disease & African Union RHD Communiqué

**DOI:** 10.21542/gcsp.2016.12

**Published:** 2016-06-30

**Authors:** Shanti Nulu, Robert C. Neely, Zeina Tawakol, Magdi Yacoub

**Affiliations:** 1 Yale University, Dept. of Medicine, Section of Cardiovascular Medicine, USA; 2 Columbia University Medical Center, Dept. of Surgery, Division of Cardiothoracic Surgery, USA; 3 Aswan Heart Centre, Magdi Yacoub Heart Foundation, Aswan, Egypt; 4 Imperial College, London, UK

The 4^th^ All-Africa Workshop on Acute Rheumatic Fever and Rheumatic Heart Disease (RHD) was held in Addis Ababa from March 4-6, 2016, hosted by the Pan-African Society of Cardiology (PASCAR) and the African Union Commission (AUC). This was a conference of expert cardiologists and cardiac surgeons who are leading RHD efforts, and included delegates from 22 African countries (Figure 1). There were also representatives from major international stakeholders, such as the World Health Organization (WHO), the World Heart Federation (WHF), as well as the philanthropic arms of the Novartis and Medtronic, both of which have active programs targeting RHD.

**Figure 1.**
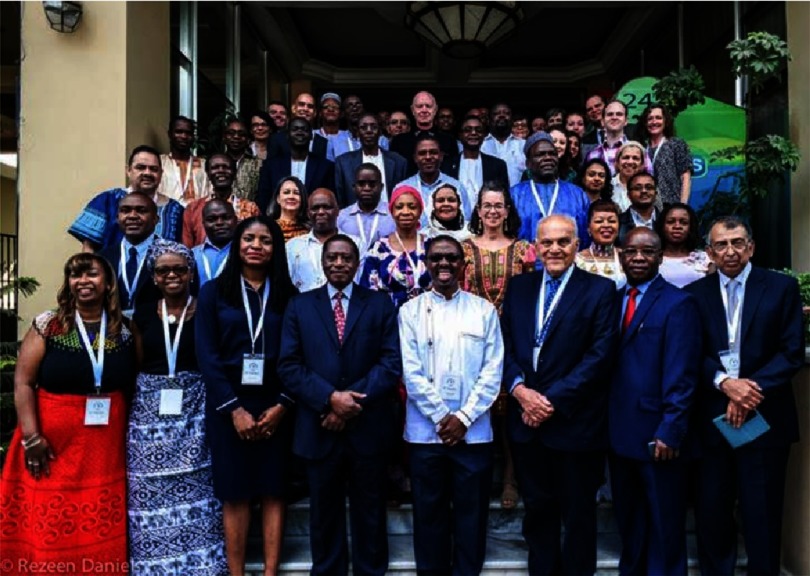
Front row left-to-right: Liesl Zuhlke, Mabvuto Kango, Ana-Olga Mocumbi, Oyere Onuma, AU Commissioner Dr Mustapha Sidiki Kaloko (Sierra Leone), Bongani Mayosi, Magdi Yacoub, John Musuku, Alaaeldin Elghamrawy. Remainder in alphabetical order: Azza Abul-Fadl; Ahmed Afifi, Sulafa Ali, Marvin Allen, Lori Allen, Michael Awoke, Fidelia Bode-Thomas, Rex Clements, Albertino Damasceno, Rezeen Daniels, Frank Edwin, Ahmed Elsayed, Mark Engel, Mario Fernandes, Paradzai Gapu, Elizabth Gatumia, Ahmed El Guindy, Prasanga Hiniduma-Lokuge, Chris Hugo-Hamman, Ali Ibrahim Toure, Neil Kennedy, Peter Lwabi, Zongezile Makrexeni, Joanna Markbreiter, Duncan Matheka, John Meda, Lwazi Mhlanti, Jeremia Mwangi, Julius Mwita, Robert Neely, George Nel, Alassane Ngaide, Shanti Nulu, Stephen Ogendo, Emmy Okello, John Omagino, Susan Perkins, Lumilla Reina, Emmanuel Rusingiza, Steven Shongwe, Maylene Shung King, Renae Stafford, Brigitta Tadmor, Zeina Tawakol, Wubaye Walegne, David Watkins and Dejuma Yadeta.

The conference commenced with an opening statement by Dr. Mustapha Sidiki Kalako, the AU Commissioner for Social Affairs, followed by presentations by the international delegates, each expounding on their current levels of engagement and resources for RHD. Next, delegates from each of the 22 countries were tasked with reporting on the status of their progress toward the “7 key actions” for intervention on RHD in Africa which were laid out a year prior, during the 3^rd^ AUC RHD conference (Table 1).

**Table 1:** 

The Addis Ababa communiqué; seven key actions to eradicate rheumatic heart disease.
1. Establish prospective RHD registries at sentinel sites in order to monitor RHD-related health outcomes, including the achievement of a 25% reduction in mortality from RHD by the year 2025
2. Ensure adequate supplies of high-quality benzathine penicillin that can be administered in the most effective manner, in order to achieve primary and secondary prevention of RHD
3. Guarantee universal access to reproductive health services for women with RHD and other NCDs
4. Decentralize technical expertise to the primary and district levels
5. Establish centres of excellence for cardiac surgery, which will deliver state-of-the-art surgical care, train African cardiac practitioners, and conduct research on cardiovascular diseases, including RHD
6. Foster multi-sectoral and integrated national RHD control programs led by the Ministry of Health, which will oversee the implementation of national RHD action plans
7. Cultivate, through a strong communication framework, partnerships with relevant stakeholders, in order to ensure the implementation of the aforementioned actions, and the connection of African RHD control measures with the emerging global movement towards RHD control

**Notes.**

ARF Acute rheumatic fever RHD Rheumatic heart disease NCD non-communicable diseases

These aims are directly derived from the 7 key barriers to RHD eradication that were identified during previous workshops, and include some structural barriers such as lack of surveillance programs, over-centralization of services, and lack of national RHD programs, as well as specific deficits, such as the supply of quality Benzathine Penicillin (BPG), poor integration with reproductive health services, and a lack of access to cardiac surgical services^1^.

This conference represents a unique moment not only for RHD, but also for global health in general. It was evident that African health leaders, working on the front lines, are in the process of reclaiming a health agenda for their own, one that reflects the actual needs of their respective populations, and one that resists the imposition of an external agenda driven by donor priorities and perceived needs. This was best represented by the key themes that emerged from this meeting, which are discussed below.

Almost all the delegates noted resistance to the RHD agenda at the government or ministry level. Many noted how competing priorities, even within the category of non-communicable diseases (NCDs), often prevailed in the funding battles of national ministries. Delegates cited two major reasons for this.

First, the lack of robust data on RHD prevalence and economic burden was suggested as a cause for its exclusion in national health agendas. Comparisons were made to other NCDs such as diabetes, where there is more data to support its urgency as a health priority. While global estimates point to a relatively low prevalence of disease, these estimates are largely statistical extrapolations from decades-old data, which relied primarily on clinical diagnostic criteria. The recent adoption of echocardiography-based screening with higher sensitivity to detect subclinical disease^2^ has shown much higher prevalence rates with the inclusion of asymptomatic children^3-5^. Given the potential economic burden from premature mortality associated with RHD among the young, the delegates uniformly agreed that further investment in research may elevate RHD as a major priority.

Second, the lack of prioritization by the WHO was widely acknowledged to be directly correlated to the agendas of national health ministries. Indeed, the relationship of many national health ministries to the WHO was described in paternalistic terms. While the WHO representative noted the existence of an RHD program within the WHO dating back to 1954, she also acknowledged the “pause” in its activities during the early 2000s, a time roughly correlated to the time at which RHD was thought to be eradicated in the West. This “pause” was essentially its de-prioritization, which represented the shifting of priorities of Western donors.

In virtually all of the 22 countries represented, the WHO role in RHD efforts by front line leaders was essentially absent. However, plans for a possible WHO Board Resolution on RHD for 2017 appear to be underway.

Indeed, there is a clear disconnect between the priorities of international global health institutions, which are focused primarily on “middle-class” NCDs such as ischemic heart disease and diabetes, and those of African health leaders who tend to the neglected diseases of the global poor. The international delegates urged for greater integration of the “7 key actions” within their own broader NCD agendas.

However a review of the WHO NCD Action Plan and the WHF Global Roadmap suggests a virtual neglect of RHD. The WHO NCD Action Plan, for instance, focuses entirely on the “big four” NCDs of cardiovascular disease, diabetes, chronic respiratory disease, and cancer and their behavioral risk factors—smoking, inactivity, dietary excess and alcohol. RHD, a disease structurally determined by poverty, inadequate access to healthcare (and antibiotics), and poor sanitation, cannot be easily integrated into this framework. As such, adhering to this framework would not address the barriers to RHD eradication observed by the African delegates.

The PASCAR RHD agenda more broadly addresses the specific structural deficiencies that have enabled RHD to thrive on the African continent. Rather than integration, international health organizations should work to elevate this framework and enable its prioritization in national health agendas. A positive step in this direction is the establishment of the RHD Action Alliance in 2015 by the WHF, together with RhEACH and Medtronic Philanthropy, which provides a platform for technical support and policy advocacy.

Two areas encompassed in the seven actions that are almost universally lacking in all represented countries are the lack of integration of RHD surveillance and treatment with reproductive services, and the lack of access to cardiac surgical services. It is thought that RHD represents an important source of maternal and perinatal mortality that is vastly under-recognized by reproductive health workers^6^. Many of the delegates who attempted to establish integrative strategies with reproductive health partners reported on the poor levels of awareness of RHD among maternal health workers. There was also universally limited access to cardiac surgical services for RHD patients, especially for those who cannot afford to pay at a private hospital.

In Egypt, the Aswan Heart Centre has served as an example of a donor-funded cardiac facility, which provides a first-world level of cardiac care free of charge, including percutaneous and surgical interventions^7^. Indeed, several other countries shared marked progress towards surgical facilities for RHD. Such examples should not be seen as exceptional cases. It is essential to keep in mind that the 7 key actions represent the entire spectrum of RHD, from primary to tertiary care, and this requires bold steps by individuals and organizations aimed at all points of the continuum.

When comparing the successes and failures of the 22 countries towards RHD eradication over the past year, countries that made the most progress were those with strong, African-led leadership. In Malawi, RHD has been included and highlighted in the national NCD plan. In Egypt, in addition to a national RHD program with 30 decentralized RHD centers, there is strong support for RHD initiatives through the efforts of the Magdi Yacoub Foundation and the Aswan Heart Centre, as well as Dr. Alaa Ghamrawy from Mahla City, whose robust research efforts which have yielded data suggesting a much higher prevalence of RHD than previously imagined. In Namibia, strong coordination of leadership at the ministry and academic levels has resulted in a national RHD registry, a national program for prevention, and outreach and decentralization efforts. Rwanda’s strong central leadership has led to robust horizontal system strengthening efforts which, although not specifically targeting RHD, have resulted in stronger resources for RHD prevention and treatment.

Education remains a vital component of prevention and treatment efforts, and this requires innovative strategies for reaching the public. The Bienmoyo Foundation in Tanzania enlisted the help of composer Danielle Williams to teach children the importance of RHD awareness through the song “Moyo Wetu” or “Our Heart”, which the PASCAR delegates had the opportunity to learn and sing (Figure 2).

**Figure 2.**
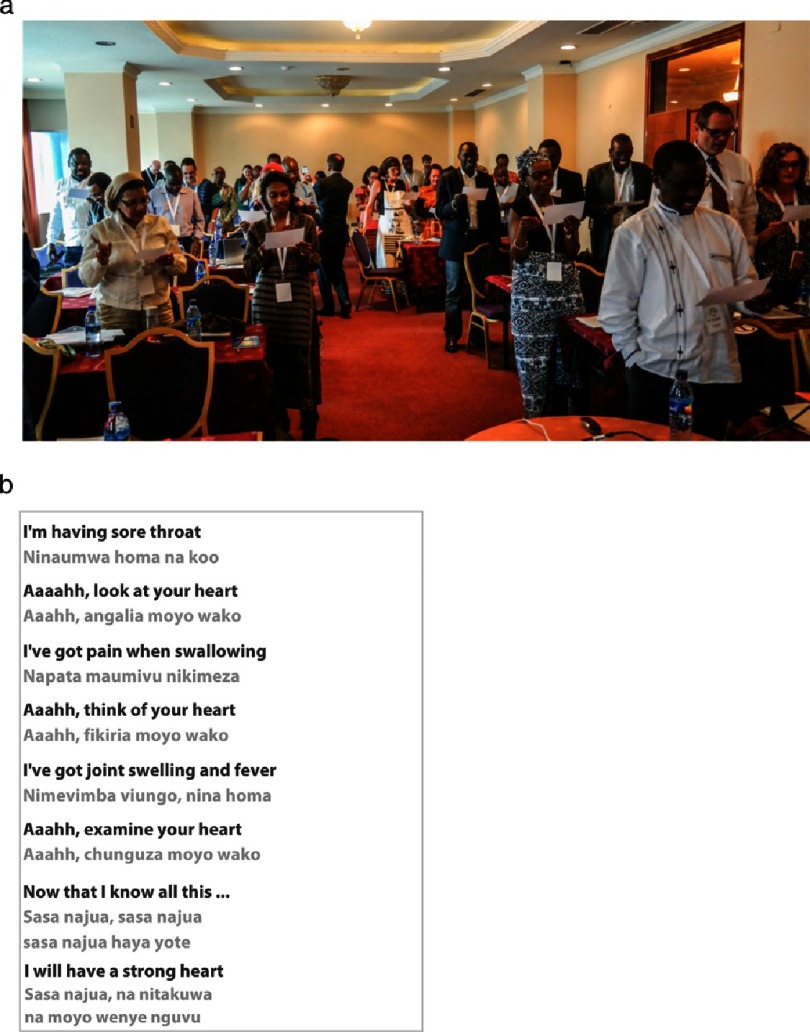
(a) Delegates sing “Moyo Wetu”, a song about rheumatic heart disease awareness taught in primary schools in Tanzania. (b) “Moyo Wetu” Lyrics by Peter Mhando, Composed by Danielle Williams.

All these themes converge to represent the voice of the front-line African health leader who resists international paternalism and reclaims an agenda that is relevant for neglected populations. This voice demands a global health equity approach which, until recently, has been overshadowed by cost-effectiveness rationalities for resource allocation. The “seven actions” agenda put forth by PASCAR is a contextually-oriented framework that has real resonance with the prevailing values of today’s global health value shifts, such as system orientation and health equity. This framework is in need of elevation by the major international health organizations, and resource prioritization to help front-line African health leaders. We eagerly anticipate next year’s follow up conference “Rheumatic Heart Disease Science and Practice—from Molecules to the Global Community”, which will be held in Cairo January 13-16^th^, 2017. We expect to build on the momentum of this year’s conference, sharing progress and emphasizing specific tasks for African leaders from diverse health care backgrounds to help make the eradication of RHD a reality.

## References

[1] Watkins D, Zuhlke L, Engel M (2016 Jan 12). Seven key actions to eradicate rheumatic heart disease in Africa: the Addis Ababa communiqué. Cardiovasc J Afr.

[2] Remenyi B, Wilson N, Steer A (2012 Feb 28). World Heart Federation criteria for echocardiographic diagnosis of rheumatic heart disease-an evidence-based guideline. Nat Rev Cardiol.

[3] Marijon E, Ou P, Celermajer DS (2007). Prevalence of rheumatic heart disease detected by echocardiographic screening. N Engl J Med.

[4] Saxena A, Ramakrishnan S, Roy A (2011). Prevalence and outcome of subclinical rheumatic heart disease in India: the RHEUMATIC (Rheumatic Heart Echo Utilisation and Monitoring Actuarial Trends in Indian Children) study. Heart.

[5] Engel ME, Haileamlak A, Zühlke L, Lemmer CE, Nkepu S, Van de Wall M, Daniel W, Shung King M, Mayosi BM (2015). Prevalence of rheumatic heart disease in 4720 asymptomatic scholars from South Africa and Ethiopia. Heart.

[6] Diao M, Kane A, Ndiaye MB, Mbaye A, Bodian M, Dia MM, Sarr M, Kane A, Monsuez J-J, Ba SA (2011). Pregnancy in women with heart disease in sub-Saharan Africa. Archives of Cardiovascular Diseases.

[7] Yacoub M, El Guindy A, Afifi A (2014). Taking cardiac surgery to the people. J Cardiovasc Translational Research.

